# Evaluation of the Design and Methodology of Applications to the Local Ethics Committee

**DOI:** 10.14744/SEMB.2021.47542

**Published:** 2021-12-29

**Authors:** Yuksel Altuntas, Zeynep Yildiz Yildirmak, Sarper Erdogan, Deniz Seckin, Asli Aksu Cerman, Hande Yapislar, Gulsum Onal, Kubra Elcioglu, Nezaket Eren, Dilek Necioglu Orken

**Affiliations:** 1. Department of Endocrinology Metabolism, University of Health Sciences Turkey, Sisli Hamidiye Etfal Training and Research Hospital, Istanbul, Turkey; 2. Department of Pediatric Hematology and Oncology, University of Health Sciences Turkey, Sisli Hamidiye Etfal Training and Research Hospital, Istanbul, Turkey; 3. Department of Public Health, İstanbul University-Cerrahpaşa, Cerrahpaşa Faculty of Medicine, Istanbul, Turkey; 4. Department of Dermatology, University of Health Sciences Turkey, Sisli Hamidiye Etfal Training and Research Hospital, Istanbul, Turkey; 5. Department of Physiology, Acibadem Mehmet Ali Aydinlar University, Faculty of Medicine, Istanbul, Turkey; 6. Department of Medical History and Ethics, Trakya University, Faculty of Medicine, Edirne, Turkey; 7. Department of Pharmacology, Marmara University Recep Tayyip Erdoğan Complex, Faculty of Pharmacy, Istanbul, Turkey; 8. Department of Biochemistry, İstanbul Yeni Yüzyıl University, Faculty of Medicine, Istanbul, Turkey; 9. Department of Neurology, Memorial Hospital, Istanbul, Turkey

**Keywords:** Case-control study, epidemiology, ethics committees, interventional studies, research misconduct; thesis studies

## Abstract

**Objectives:**

Proposals for scientific studies must have an original hypothesis and the appropriate design and methodology to test the premise.

**Methods:**

This study is an evaluation of the suitability of applications submitted to a local ethics committee (EC) and the rate of publication of that research.

**Results:**

A total of 899 files submitted for EC approval were retrospectively assessed. The EC found that the description of the methods in 44% of the applications was inaccurate, and that this type of error was most often seen in submissions from the surgical branch. In all, 52% of the applications for which we were informed about their final status were not published.

**Conclusion:**

The results suggest that improved training in epidemiology is required to reduce the number of application errors and that new regulations could help to motivate healthcare personnel to conduct scientific research and publish their findings.

Medical research ethics boards have a complex role with a mission that serves the public. In addition to an obligation to examine the scientific merit and soundness of proposed biomedical research a primary duty is to ensure compliance with ethical principles that protect the rights of human and animal subjects, to monitor research studies, and to provide formal assessment opinions.^[1]^ The legal foundation of the current medical research ethics committees (ECs) active in Turkey is the Regulation on Drug Research published in 1993. The regulation directed that local ECs were to be established at universities and teaching hospitals that would be the sites of research, as well as a central EC within the Ministry of Health.^[2]^ The Regulation on Clinical Trials, which had the objective of ensuring compliance with European Union standards and protecting the rights of individuals participating in clinical trials, came into force in 2008 and required approval from one of these committees for the conduct of such research. ECs consist of a minimum of 7 and a maximum of 15 volunteer members. The present minimum qualifications for the membership of an EC for clinical research are:

1. Specialist physicians who have participated in international clinical research conducted according to the rules of good clinical practice and who are preferably selected from different specialties
2. A person holding a doctoral or medical residency degree in pharmacology
3. A person who has a PhD in biostatistics or a public health specialist, or a medical doctor who has a PhD in public health
4. A biomedical engineer or specialist, or, if not available, a biophysicist or physiologist
5. A jurist
6. A non-healthcare professional (members of the public)
7. If available, a person who has a PhD in medical ethics or deontology (ethicists).

At least three of the committee members are to be selected from outside the institution where the EC secretariat is located. Among its responsibilities, the EC is charged with an analysis of potential benefits, harms, and risks of a study; determining whether the research is based on sound scientific data and an original hypothesis; and assessing the protocol and the methods to be used, including obtaining the voluntary consent of any human participants. These constitute essential minimum requirements for the evaluation of a research application.^[3]^ Following this preliminary assessment, the applicants are sent a form advising them of any issues to be revised or corrected. Files resubmitted with the required amendments are re-evaluated by the EC.

The University of Health Sciences Turkey, Istanbul Şişli Hamidiye Etfal Training and Research Hospital EC is 1 of 126 ECs currently established in Turkey and has been in service since 2009. The group evaluates approximately 150 applications each year. The purpose of this study was to evaluate the design and methods described in EC applications from our institution that were reviewed by the committee and the rate of publication of these studies.

## Methods

The Şişli Hamidiye Etfal EC meets every 2 weeks and has a total of 11 members: 5 specialists of clinical fields, 1 public health physician, 1 pharmacologist, 1 physiologist, 1 deontologist, 1 jurist, and 1 member not affiliated with a health institution. The applications are first evaluated before the meeting by the member serving as rapporteur and then presented to the full membership of the EC at the meeting.

This study was a retrospective examination of 912 files that were submitted for approval during the 6-year period of 2012–2018. Thirteen files from external centers were excluded from this review. The year of application; the subject of the study; the clinic where the responsible researcher was employed; the purpose of the study, defined as individual research or a specialized thesis study; and the research method specified in the application were recorded. The nature and details of all requests for revision issued by the EC were noted, including comments related to the application form, participant consent, protocol content, and the statistical methods described. The EC members met outside of regular meetings and evaluated the appropriateness of the research method defined by the applicant. The directors of the 31 clinics where the research applicants worked were provided with the names of the authors and the title and year of the study and asked to reply with details of the publication status of these studies. A response was received from 28 of 31 clinics that were requested to provide information. Inclusion of the research in the TR Index of the Turkish Academic Network and Information Center (ULAKBIM), a research and development facility institute of the Scientific and Technological Research Council of Turkey (TUBITAK); the Science Citation Index (SCI); and/or the Science Citation Index Expanded (SCIE) was recorded.

## Results

A total of 899 files were included in the study (Table 1). A mean of 152 applications were reviewed per year during the study period. In all, 67.8% of the observational studies were descriptive and 21.4% were case-control studies. Cross-sectional studies accounted for 5.8% and cohort studies for 4.9%.

**Table 1.** Distribution of study approval applications by year

**Year**	**Number**	**%**
2012	134	14.9
2013	144	16.0
2014	129	14.3
2015	176	19.6
2016	128	14.3
2017	188	20.9
Total	899	100.0

Out of 899 application files 352 had a definition of the method they planned to use. The EC found 66% of them described in the application form to be accurate (Table 2).

**Table 2.** EC opinion and consistency with the method specified in the application

**Application**	**Method found to be appropriate by the EC**						**Total**
	**Descriptive**	**Cross-sectional**	**Case-control**	**Cohort**	**Methodological**	**Interventional**
Descriptive							
Count	152	3	23	3	2	32	215
%	70.7	1.4	10.7	1.4	0.9	14.9	100.0
Cross-sectional							
Count	33	10	14	4	2	4	67
%	49.3	14.9	20.9	6.0	3.0	6.0	100.0
Case-control							
Count	2	0	10	0	0	2	14
%	14.3	0.0	71.4	0.0	0.0	14.3	100.0
Cohort							
Count	1	0	0	2	0	1	4
%	25.0	0.0	0.0	50.0	0.0	25.0	100.0
Methodological							
Count	0	0	0	0	6	0	6
%	0.0	0.0	0.0	0.0	100.0	0.0	100.0
Interventional							
Count	3	0	2	0	0	41	46
%	6.5	0.0	4.3	0.0	0.0	89.1	100.0
Total							
Count	191	13	49	9	10	80	352
%	54.3	3.7	13.9	2.6	2.8	22.7	100.0

The interventional method was correctly described in 89.1% of the applications, 71.4% of those described as case-control studies were correctly identified, and 70.7% of descriptive studies. The cross-sectional study description was the least accurate, with only 14.9% concurrence with the definition.

When errors in defining the type of study were examined in 899 files according to discipline, the correct description was observed in 44.4% of the internal medicine applications, 12.1% of those from the surgical sciences, and 16.2% of those representing the basic sciences. A total of 30.9% were correctly defined, which was statistically significant (Chi-square test; p<0.05) (Table 3).

**Table 3.** Errors in defining the study type according to disciplines

**Branch**	**Description**		**Total**
	**Correct**	**Incorrect and undefined**	
Internal medicine			
Count	231	291	522
%	44.3	55.7	100.0
Surgical sciences			
Count	41	299	340
%	12.1	87.9	100.0
Basic sciences			
Count	6	31	37
%	16.2	83.8	100.0
Total			
Count	278	621	899
%	30.9	69.1	100.0

**Figure 1.**
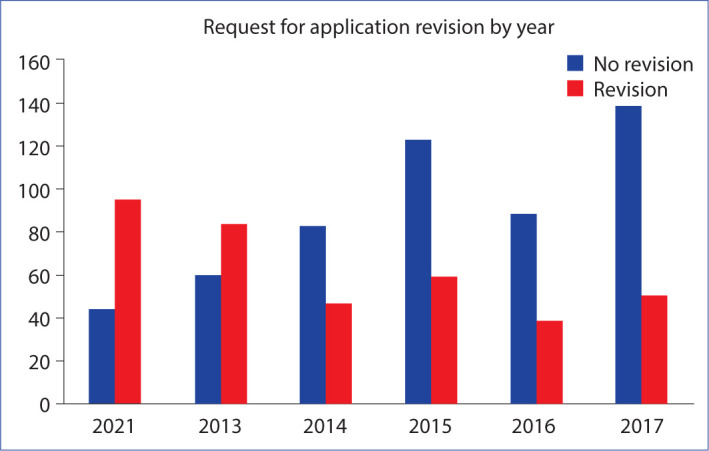
Request for application revision by year.

Correction was requested in 41% of the application files submitted to the EC during the period evaluated. The request for revision was related to the application form in 7.5% of the proposals, 13.6% had consent inadequacies, 15.1% revealed weaknesses in the research protocol, and 4.8% needed modifications to the statistical analysis methods. It was observed that there was a decrease in EC requests for revisions to applications over the study period: 68% of applications required amendment in 2012, while the ratio decreased to 26.5% in 2017 (Fig. 1).

Examination of the distribution of requests for modification by branch revealed that 37.7% of the requests were applications from internal medicine, 48.2% from the surgical sciences, and 18.9% from the basic sciences, and the difference was statistically significant (Chi-squared test; p<0.05) (Table 4).

**Table 4.** Distribution of requests for application revision by disciplines

**Branch**	**Correction**		**Total**
	**(+)**	**(–)**	
Internal medicine			
Count	197	325	522
%	37.7	62.3	100.0
Surgical sciences			
Count	164	176	340
%	48.2	51.8	100.0
Basic sciences			
Count	7	30	37
%	18.9	81.1	100.0
Total			
Count	368	531	899
%	40.9	59.1	100.0

Of the evaluated studies, 49 (16.8%) were research studies involving drugs, while 26 (8.9%) were device studies. The number of drug investigations decreased over the study period (Table 5).

**Table 5.** Distribution of drug and device studies by year

**Year**	**Type of study**			**Total**
	**Drug**	**Device**	**Other**	
2012	17	3	22	42
	40.5%	7.1%	52.4%	100.0%
2013	11	6	31	48
	22.9%	12.5%	64.6%	100.0%
2014	5	0	40	45
	11.1%	0.0%	88.9%	100.0%
2015	5	6	45	56
	8.9%	10.7%	80.4%	100.0%
2016	6	5	34	45
	13.3%	11.1%	75.6%	100.0%
2017	5	6	44	55
	9.1%	10.9%	80.0%	100.0%
	49	26	216	291
Total	16.8%	8.9%	74.2%	100.0%

During the period evaluated, 535 of the applications were classified as individual research studies (58.7%) and 368 were thesis studies (40.4%). The type was not specified in 9 cases.

More than half (58%) of the 899 studies were from the branch of internal medicine, and there were only 37 applications (4%) from the basic sciences, which was a statistically significant difference in the number of applications by source (Pearson Chi squared; p<0.05). Most of the proposals were observational studies (64.5%), while 32.3% were interventional studies and 3.2% were methodological studies.

Interventional studies were more common (55.9%) in applications from surgical specialties, while only 19% and 2.7% were from internal medicine and the basic sciences, respectively. The frequency of observational studies from the basic sciences and internal medicine was 81.1% and 77.6%, respectively. The frequency of methodological studies in the basic sciences was statistically significantly greater than that of other branches (16.2%).

There was no information about the publication status of 447 projects submitted to the EC, while we were informed that 217 of the 452 remaining studies were published. Of those, 50.7% were published in SCI and SCIE journals, 49.3% were published in journals indexed in databases other than SCI and SCIE, or in the ULAKBIM TR Index. Among the 217 studies published in SCI and SCIE journals, 59.6% were individual studies and 28.1% were thesis studies (Table 6).

**Table 6.** Distribution of publications by type

	**SCI + SCI-E**	**Other than SCI + TR Index**	**Total**
Individual	90	61	151
	59.6%	40.4%	100.0%
Thesis	18	46	64
	28.1%	71.9%	100.0%
Unknown	2	0	2
	100.0%	0.0%	100.0%
Total	110	107	217
	50.7%	49.3%	100.0%

## Discussion

The mean of 152 applications reviewed per year by the Şişli Hamidiye Etfal EC is above average. Alev et al.^[4]^ reported in 2015 that one-third of the 180 Turkish universities had an EC and only 39% of those evaluated 21 or more applications each year.

As expressed by Zain et al.,^[5]^ the main purpose of writing and publishing a study is to disseminate research findings and to share new knowledge with other researchers in the field. In contrast, a primary motivation for carrying out a study in this country has been to meet the requirements for promotion to an associate professorship position, which include consideration of the quantity of scientific publications.

Thesis studies, which are required to complete a residency, also constitute another major group of applications. A study related to EC applications for non-invasive studies submitted by a private foundation university found that the percentage of individual study and residency thesis applications was 36.5–45.5% and 5.3–6.9%, respectively.^[3]^ However, Şişli Hamidiye Etfal is a training and research hospital, and hospitals of this category generally graduate many specialists each year, unlike foundation universities. This could explain the large representation (40.4%) of thesis studies among our applications.

Of the 899 applications reviewed by our EC, more than half (58%) originated from the internal medicine branch and only 4% came from basic science disciplines. This was likely due to the fact that microbiology and biochemistry are the only basic science divisions active in our hospital, while there are 17 internal and 11 surgical divisions.

The applications submitted by the surgical branch were almost all interventional studies without control groups. Since the primary methods of surgical therapy are invasive, this is naturally reflected in the applications. As an interventional study type, randomized controlled trials (RCTs) are the gold standard in terms of study design, but this was not the preference of our surgeons. It may be related to the observation of Cao et al.^[6]^ that in the surgical setting, conducting RCTs can often be unethical or logistically impossible. They suggested that case-control studies should be the primary study design used in surgical research when RCTs are not feasible.^[6]^ The applications from surgeons also received the greatest number of requests for correction from the EC. Nearly all clinicians, including surgeons in research hospitals, work according to the “publish or perish” principle, which contributes the additional difficulty of acting as a researcher to the work of being a treating physician. Along with the paperwork and examination of the patient, surgeons, unlike other physicians, also perform interventions in order to treat their patients. The long duration of operations, the extra responsibilities and sensitivities of critical operations, such as oncological surgery, add to their workload both physically and mentally. This often decreases the time spent preparing a research project, collecting and analyzing the data, and writing a scientific report. We are of the opinion that this shortage of time could be the reason for the greater frequency of mistakes seen in surgical study applications.

Our training and research hospital is a tertiary referral center that treats complicated cases from all over the country. These are usually cases that could not be treated sufficiently in secondary care hospitals. The best epidemiological method to investigate these types of cases is a case-control study. The rare diseases seen in our hospital, sometimes with long incubation periods, could explain why the case-control study design was the most preferred method.^[7]^ A case-control study is easy to conduct and inexpensive relative to other methodologies. Case-control is the most common study design not just in our hospital, but worldwide.^[8]^ Young researchers in particular, like those at our hospital who seek a promotion to an associate professorship position, can find the opportunity to carry out several studies in a relatively short time period.

However, undergraduate medical students are often not very willing to learn and absorb the fundamental principles of epidemiology. Moffat et al.^[9]^ reported that many students regarded epidemiology as dry and boring and wanted practical and clinically relevant instruction. In Turkey, the fundamentals of epidemiology are given in public health classes. Although the class time devoted to epidemiology is limited, student absenteeism for these relatively few lectures that provide the basics of the field is a problem. The effects have been visible in application errors, particularly in the methodology section. In some cases, the name of the methodology to be used was not consistent with the methodology described.

A lack of willingness by the applicant to revise the file can cause a delay in the evaluation process of the EC, and sometimes there are disagreements between the applicants and committee members.^[10]^ This reluctance of researchers could have a direct correlation with their knowledge or appreciation of epidemiology; those who are uninterested in epidemiology may be making more mistakes when writing up a project. Suzuki et al.^[11]^ reported that approximately half of all examined proposals lacked a clearly written “Background” section that defined the study rationale and design. The principles of epidemiology should guide the researcher’s definition of the hypothesis based on the research question and the study design most suited to answer that question. The study design directs how the investigation is conducted.^[12]^

A belief that the EC evaluates only certain ethical issues may also contribute to weak applications. In fact, the EC is responsible for the entire proposal. An application that does not have a sound scientific basis, proper methodology, and the necessary details cannot be evaluated as ethical.^[13]^ Some of the revisions requested after the evaluation process suggest the need for additional training of researchers about preparing a study project.^[14]^ In our study, 41% of the applications were returned with a request for correction, while Yildirim^[14]^ reported a total of 76% of the applications received. A preliminary review upon submission performed by an experienced administrative secretary can help to reduce the return rate, as in our case. Furthermore, we found that the return rate for requested corrections has decreased and that the quality of the application files has improved. The evaluations of the EC may have played an informative role.

In Turkey, authorship of publications in internationally indexed medical journals is required for promotion in an academic career. Therefore, this is of great importance to applicants who hope to pursue an academic career. It is not the same with thesis studies. Since a small percentage of residents have a chance to be employed in academia, not all thesis studies were submitted to a medical journal for publication.

## Conclusion

The Şişli Hamidiye Etfal Training and Research Hospital EC accepts and reviews more applications per year than most ECs in Turkey and could be regarded as an exemplar in some respects.

The most commonly used methodology among the applications was a case-control study, which is quick, inexpensive, and appropriate for rarely seen cases. The surgical specialties more often use an interventional study approach, which is inherent in the nature of the discipline.

The errors made by the applicants when preparing the study proposal indicated that there is a significant need to improve epidemiology training for healthcare personnel who are going to perform scientific research.

In contrast to a general rule that the number of basic science studies exceeds the number of applied science studies, the common lack of most basic science disciplines at training and research hospitals led to a low percentage of basic science studies brought to our EC.

The percentage of studies published in an indexed journal was low considering the total number of approved applications. Contrary to the expectations of some, the primary motivation for carrying out a scientific study is often not the personal interest of the researcher, but rather the need to meet requirements for academic promotion or residency. Thus, the decisions of politicians who regulate these areas in Turkey could easily have an impact on the number of research applications. Another possible reason of low percentage of publication rate is having financial support from the hospital management only to have a local EC approval. This may result in applications without starting the study.

### Disclosures

**Ethics Committee Approval:** The retrospective study.

**Peer-review:** Externally peer-reviewed.

**Conflict of Interest:** None declared.

**Authorship Contributions:** Concept: Y.A., Z.Y.Y., S.E., D.N.Ö.; Design – Y.A., Z.Y.Y., S.E., D.N.O.; Supervision – S.E., Y.A., Z.Y.Y., D.N.O.; Materials – Y.A., Z.Y.Y., S.E., D.N.O., A.A.C., H.Y., K.E., G.O., N.E.; Data collection and/or processing – Y.A., Z.Y.Y., S.E., D.N.O., A.A.C., H.Y., K.E., G.O., N.E.; Analysis and/or interpretation – Y.A., Z.Y.Y., S.E., D.N.O., A.A.C., H.Y., K.E., G.O., N.E., D.S.; Literature search – Z.Y.Y., S.E., D.N.O.; Writing – Y.A., Z.Y.Y., S.E., D.N.O., A.A.C., D.S.; Critical review – S.E., Z.Y.Y., D.N.O., Y.A.

## References

[1] Hakeri H (2013). Tıp Hukuku..

[2] Somer P, Vatanoğlu E (2013). Ethics committees within the scope of the regulation of clinical research.. Marmara Üniversitesi Hukuk Fakültesi Hukuk Araştırmaları Dergisi.

[3] Ilbars H, Bebitoglu BT (2018). How to get ethics committee approval for clinical trials in Turkey?. North Clin Istanb.

[4] Alev B, Genç FN (2015). A review of the university ethics committee in Turkey.. Akdeniz İİBF Journal.

[5] Zain S, Ab-Rahman MS, Ariffin AK, Zahrim A, Nor JM, Zain MFM (2011). Motivation for research and publication: Experience as a researcher and an academic.. Procedia Social and Behavioral Sciences.

[6] Cao AM, Cox MR, Eslick GD (2016). Study design in evidence-based surgery: What is the role of case-control studies?. World J Methodol.

[7] Çakır B (2003). Medical research style: Basic features, benefits and limitations, possible sources of error.. Thorax Society.

[8] Schulz KF, Grimes DA (2006). The Lancet Handbook of Essential Concepts in Clinical Research..

[9] Moffat M, Sinclair HK, Cleland JA, Smith WC, Taylor RJ (2004). Epidemiology teaching: student and tutor perceptions.. Med Teach.

[10] Shetty YC, Marathe PA, Billa GV, Nambiar CP (2012). A study to assess completeness of project application forms submitted to Institutional Ethics Committees (IEC) of a tertiary care hospital.. Perspect Clin Res.

[11] Suzuki M, Sato K (2016). Description and evaluation of the research ethics review process in Japan: Proposed measures for improvement.. J Empir Res Hum Res Ethics.

[12] Chatburn RL (2017). Basics of study design: Practical considerations (From the "Biostatistics and Epidemiology Lecture Series, Part 1"). Cleve Clin J Med.

[13] Meral İ, Aşti T, Soysal Ö, Yıldırım N, Özçelk S, Aydın T (2019). The inadequacies and the most-made errors in project application files submitted to a university ethics committee for non-invasive studies.. Turkiye Klinikleri J Med Ethics..

[14] Yıldırım G (2016). Evaluation of letters of applications given to the non-interventional clinical research ethics committee. Turkish Journal of Bioethics.

